# Large surgically resected leiomyosarcoma of the liver: a case report

**DOI:** 10.1186/s40792-020-00934-6

**Published:** 2020-07-09

**Authors:** Takashi Maeda, Kyohei Yugawa, Nao Kinjo, Daisuke Imai, Kensaku Sanefuji, Koto Kawata, Shinichiro Ikeda, Keitaro Edahiro, Kazuki Takeishi, Tomohiro Iguchi, Noboru Harada, Mizuki Ninomiya, Shohei Yamaguchi, Kozo Konishi, Shinichi Tsutsui, Hiroyuki Matsuda

**Affiliations:** 1 Department of Surgery, Hiroshima Red Cross Hospital and Atomic-bomb Survivors Hospital, 1-9-6 Senda-machi, Naka-ku, Hiroshima 730-8619, Japan; 2 Department of Surgery and Science, Graduate School of Medical Sciences, Kyushu University, 3-1-1 Maidashi, Higashi-ku, Fukuoka 812-8582, Japan

**Keywords:** Leiomyosarcoma, Liver tumor, Immunohistochemistry

## Abstract

**Background:**

Primary hepatic leiomyosarcoma (PHL) is an extremely rare type of tumor. We herein report a case of a large surgically resected leiomyosarcoma of the liver.

**Case presentation:**

A 69-year-old man with a feeling of epigastric compression was referred for examination of an abdominal mass. He had no history of liver disease or alcohol abuse. Liver function tests indicated Child-Pugh class A. Tumor markers were negative. Computed tomography (CT) and magnetic resonance imaging (MRI) revealed a relatively well-contrasted 12 × 11 × 8 cm tumor with well-defined boundary replacing the lateral segment of the liver alongside multiple intrahepatic metastases. Several nodules up to 12 mm were found in both lungs, suggestive of metastasis. SUVmax of the liver mass and lung tumor in positron emission tomography were 10.4 and 1.5, respectively. Hepatocellular carcinoma was primarily suspected. Lateral segmentectomy of the liver was performed to confirm diagnosis and prevent tumor rupture. Macroscopically, the lateral segment of the liver had been replaced by a lobular or multinodular tumor with a maximum diameter of 15 cm. In pathological findings, the tumor consisted of bundle-like proliferation of complicated banding spindle-like cells with clear cytoplasm, accompanied by storiform pattern and compressed blood vessels. Nuclear fission images were observed in 8/10 HPF. Partial necrosis was present, with associated venous invasion and intrahepatic metastasis. Immunohistochemical staining for tumor cells revealed desmin, α-smooth muscle actin (αSMA), and h-caldesmon were all positive, informing a final diagnosis of PHL. The postoperative course was uneventful, and he was discharged on the 12th postoperative day.

**Conclusions:**

PHL is a rare malignant disease with relatively poor prognosis. To confirm a diagnosis of PHL, immunohistochemical analysis as well as histopathological findings is important. The preferred treatment is surgical resection, sometimes in combination with adjuvant chemotherapy and radiotherapy. Further studies are needed to elucidate and better understand this uncommon clinical entity.

## Background

Primary hepatic leiomyosarcoma (PHL) is an extremely rare tumor, accounting for 0.2–2% of all primary hepatic malignancies [1–6]. PHL usually arises from intrahepatic vascular structures, bile ducts, or ligamentum teres [7–9]; however, the underlying pathogenetic mechanisms have not yet been identified. Clinical manifestations of PHL are nonspecific, and tumors are generally asymptomatic until they significantly increase in size [7, 8]. Herein, we report a case of a 69-year-old male patient with a large surgically resected PHL.

## Case presentation

**Fig. 1:**
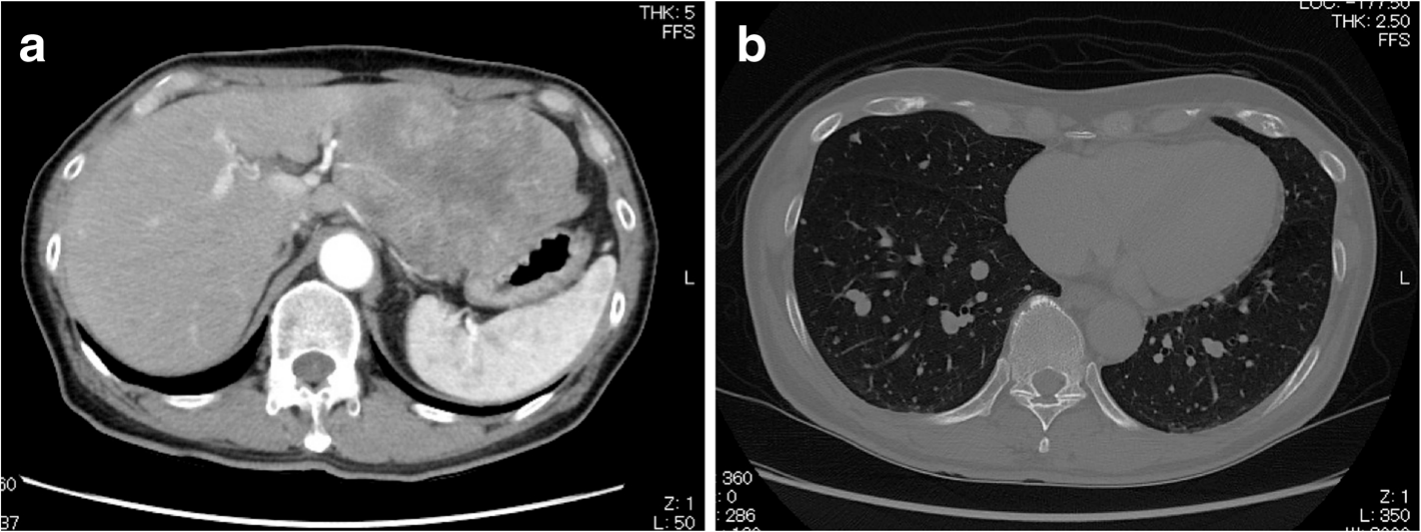
CT demonstrated **a** a relatively well-contrasted 12 × 11 × 8 cm tumor with well-defined boundary replacing the lateral segment of the liver. Contrast-enhanced CT revealed a heterogeneous and peripheral enhancement on early phase. **b** Several nodules up to 12 mm were observed in both lungs, suggestive of metastasis

**Fig. 2:**
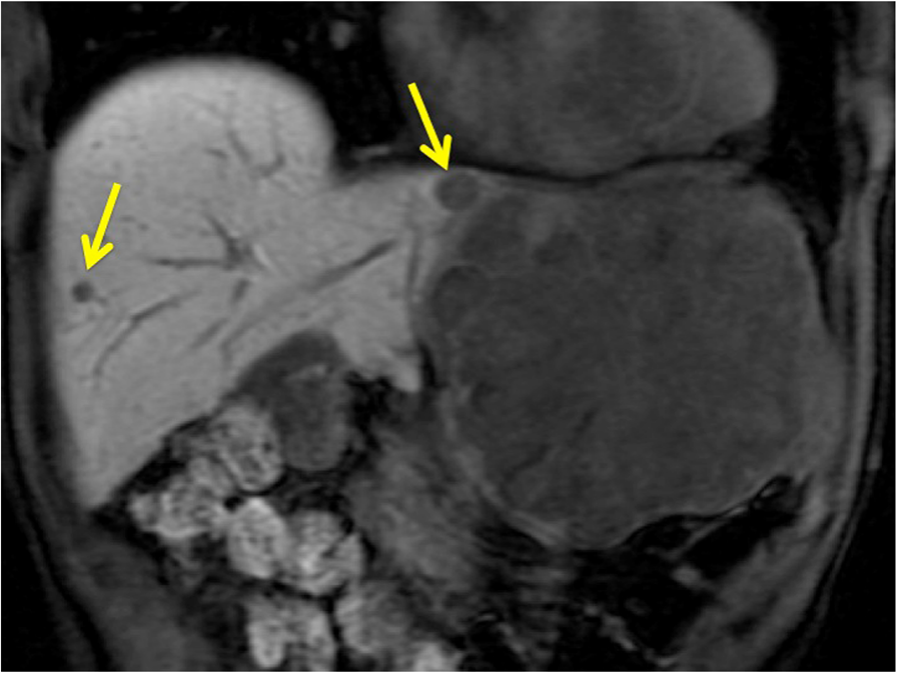
MRI demonstrated a large tumor and multiple intrahepatic metastases (arrow)

**Fig. 3:**
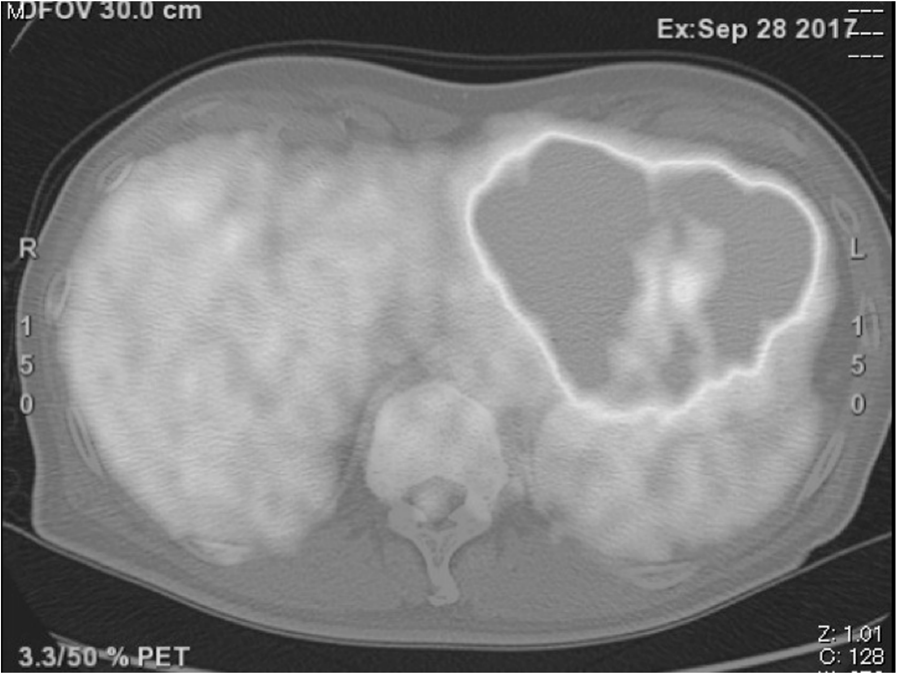
SUVmax of the liver mass in PET was 10.4. No abnormal accumulation was observed in the lymph nodes, bones, or peritoneum

**Fig. 4:**
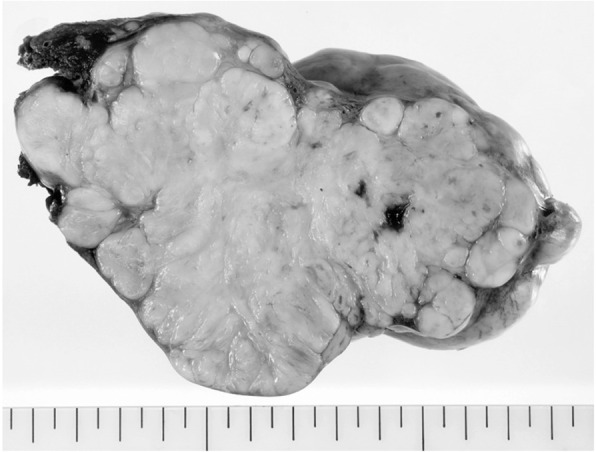
Macroscopically, the lateral segment of the liver had been replaced by a lobular or multinodular tumor with a maximum diameter of 15 cm

**Fig. 5:**
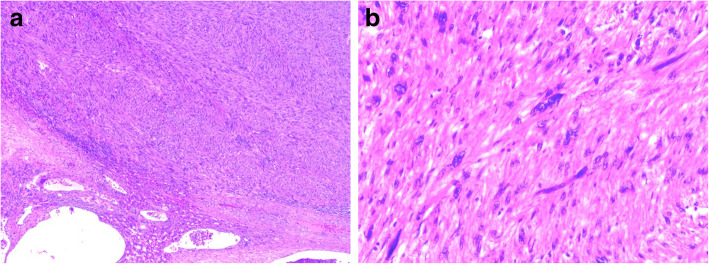
In pathological findings, **a** the tumor consisted of bundle-like proliferation of complicated banding spindle-like cells with clear cytoplasm, accompanied by storiform pattern and compressed blood vessels. **b** Various degrees of nuclear atypia were observed, and nuclear fission images were demonstrated in 8/10 HPF

**Fig. 6:**
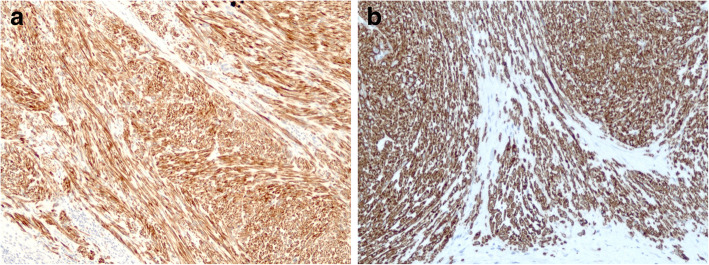
On immunohistochemical staining, **a** desmin and αSMA and **b** h-caldesmon were diffusely positive, while c-kit, S100, CD34, CAM5.2, EMA, CD163, and DOG1 were negative

A 69-year-old man with a feeling of epigastric compression was referred to our hospital for examination of an abdominal mass. His past medical history included hyperlipidemia, pulmonary emphysema, and bronchial asthma. He had no history of liver disease, blood transfusion, tattooing, or alcohol abuse. He had smoked 30 cigarettes per day for 35 years. The patient’s laboratory findings on admission were as follows: white blood cell count, 7500/μl; hemoglobin, 13.7 g/dl; platelet count, 21.1 × 10^4^/μl; C-reactive protein, 0.5 mg/dl; total protein, 6.7 g/dl; serum albumin, 4.0 g/dl; UN, 11.1 mg/dl; serum creatinine, 0.7 mg/dl; glucose, 86 g/dl; and HbA1c, 5.4%. Liver function tests revealed total bilirubin, 0.5 mg/dl; serum aspartate aminotransferase, 21 IU/l; serum alanine aminotransferase, 14 IU/l; serum glutamyltransferase, 83 IU/l; prothrombin time, 80.8%; and indocyanine green retention rate at 15 min, 8.6%, and indicated Child-Pugh class A. Antibody tests were negative for both hepatitis B surface antigen and hepatitis C virus. The following tumor markers were all within normal ranges: α-fetoprotein was 5.7 ng/ml (normal range < 10 ng/ml), PIVKA-II was 18 mAU/ml (normal range < 40mAU/ml), carcinoembryonic antigen was 1.5 ng/ml (normal range < 5.0 ng/ml), carbohydrate antigen 19-9 was 4.1 ng/ml (normal range < 37.0 ng/ml), and sIL-2R was 362 U/ml (normal range 121–613 U/ml). Abdominal computed tomography (CT) (Fig. 1) and magnetic resonance imaging (MRI) (Fig. 2) revealed a relatively well-contrasted 12 × 11 × 8 cm tumor with a well-defined boundary replacing the lateral segment of the liver alongside multiple intrahepatic metastases. In addition, several nodules up to 12 mm were found in both lungs, suggesting metastasis. Contrast-enhanced CT revealed a hypodense tumor on plain scans, with heterogeneous and peripheral enhancement on early phase and delayed washout on late phase. MRI revealed a homogenous hypointense tumor on Tl-weighted images and a hyperintense one in T2-weighted images without encapsulation. On Gd-EOB-DTPA-enhanced MRI, the tumor was heterogeneously enhanced during the early phase and weakly enhanced during the late phase. SUVmax of the liver mass and the lung tumor in positron emission tomography (PET) were 10.4 and 1.5, respectively (Fig. 3). No abnormal accumulation was observed in the lymph nodes, bones, peritoneum, or any other sites. Upper and lower gastrointestinal endoscopic examination detected no abnormal findings. Hepatocellular carcinoma was primarily suspected; however, intrahepatic cholangiocarcinoma or mixed hepatocellular and cholangiocarcinoma were other possible diagnoses. Lateral segmentectomy of the liver was performed to confirm diagnosis and prevent tumor rupture. Operation time was 104 min, with a bleeding volume of 240 ml. Macroscopically, the lateral segment of the liver was replaced by a lobular or multinodular tumor with a maximum diameter of 15 cm (Fig. 4). In pathological findings, the tumor consisted of bundle-like proliferation of complicated banding spindle-like cells with clear cytoplasm, accompanied by storiform pattern and compressed blood vessels (Fig. 5). Hydropic change and hyalinization were observed. Various degrees of nuclear atypia were observed, and nuclear fission images were demonstrated in 8/10 high-power field. Partial necrosis was present, with associated venous invasion and intrahepatic metastasis. Immunohistochemical staining for tumor cells revealed desmin, α-smooth muscle actin (αSMA), and h-caldesmon to be diffusely positive, while c-kit, S100, CD34, CAM5.2, EMA, CD163, and DOG1 were negative (Fig. 6). Ki-67 labeling index of the main tumor was 70%. These findings suggested differentiation into the smooth muscle, informing a final diagnosis of leiomyosarcoma of the liver. No other neoplastic lesion was detected on image examinations such as gastrointestinal endoscopy, CT, MRI, and PET, so the tumor was considered to be primary, not metastatic liver tumor. Postoperative course was uneventful, and he was discharged on the 12th postoperative day. Four weeks after surgery, systemic chemotherapy with doxorubicin (75 mg/m^2^) was commenced for metastatic tumors of the liver and lung.

## Discussion

PHL is an extremely rare tumor, accounting for 0.2–2% of all primary hepatic malignancies [1–6]. PHL usually arises from intrahepatic vascular structures, bile ducts, or ligamentum teres [7–9]. The underlying pathogenetic mechanisms of the disease have not yet been identified; however, it is reportedly associated with AIDS, Epstein-Barr virus, Hodgkin’s lymphoma, immunosuppression after organ transplantation, and HCV-related liver cirrhosis [4, 7, 10, 11].

PHL diagnosis is challenging due to its nonspecific symptoms and lack of serological markers. Clinical manifestations and laboratory and imaging examinations have limited value for diagnosis and differential diagnosis. Some patients may present with a wide spectrum of symptoms, including abdominal pain, abdominal mass, weight loss, nausea, vomiting, anorexia, abdominal distension, jaundice, and, rarely, acute intra-abdominal bleeding secondary to tumor rupture [4, 7]. PHL is often asymptomatic until it increases significantly in size, resulting in nonspecific symptoms, or is unexpectedly found during physical examination [7, 8]. Tumor size at diagnosis varies greatly, with the largest being 30 cm in diameter. The mean and median diameters for all reported PHLs are 10.3 cm and 9.1 cm, respectively [4]. In this case, the tumor was detected as a large mass, 15 cm in diameter, by detailed examination for a feeling of epigastric compression.

Wide ranges of data exist in the literature regarding radiological and histological findings of PHL. Ultrasonography generally shows a hypoechoic mass or a mass with heterogeneous echogenicity [4]. CT findings of PHL generally describe enlarged, heterogeneous, hypodense masses with peripheral enhancement and evidence of central necrosis [4, 7, 8, 12, 13], whereas MRI usually displays homogeneous or heterogeneous hypointensity tumors on T1-weighted images and hyperintensity on T2-weighted images [4, 7]. Ferrozzi et al. [12] reported that hemorrhagic necrosis is likely to occur inside the tumor when the tumor grows, and the tumor may show cystic degeneration after necrosis. In this case, the main tumor was large, with heterogeneous and peripheral enhancement on both CT and MRI, suggesting central necrosis of the tumor. Several studies reported that SUVmax of the tumor was correlated with the tumor size, TNM staging, histology subtype, and so on [14–16]. SUVmax of the main tumor in this case was 10.4, much higher than those of metastatic tumor of the liver and lung, which may reflect tumor size. Definitive diagnosis of PHL mainly depends on pathological and immunohistochemical examinations. Histopathological diagnosis of PHL is based on the presence of uniform and diffuse infiltrates of spindle-shaped cells with hyperchromatic nuclei, and presence of mitotic figures [7, 8]. Positive immunohistochemistry reactions for αSMA, desmin, and vimentin and negative reaction for cytokeratin, S100 protein as well as α-fetoprotein, CD34, CD117, and hepatocytes are used to further confirm diagnosis [4, 7–11, 17]. In this case, tumor cells were positive for desmin, αSMA, and h-caldesmon and negative for c-kit, S100, CD34, CAM5.2, EMA, CD163, and DOG1, supporting the diagnosis of PHL.

Regarding therapeutic options for PHL, hepatic resection is considered the only potentially curative treatment for tumors without distant metastases [4–8]. Matthaei et al. [18] previously reported three cases with > 10 years survival after hepatectomy. Liver transplantation outcomes for PHL have remained controversial to date [19, 20]. Furthermore, the efficacies of chemotherapy and radiotherapy for PHL have not been confirmed. Moreover, the role of adjuvant chemotherapy/chemoradiotherapy in PHL is not well defined. Some authors have previously reported the addition of adjuvant chemotherapy consisting of various drug combinations [2, 4, 8, 13, 17, 21, 22]. Chemotherapeutic regimens administrated included the following: folinic acid, fluorouracil, irinotecan, and bevacizumab [17]; mitoxantrone, cisplatin, and fluorouracil [22]; and ifosfamide and mesna [13]. Adjuvant chemotherapy using doxorubicin and ifosfamide seems to slow progression and help to prolong survival after complete resection [9]. Currently, no effective treatments have been reported for unresectable PHL. Fujita et al. [23] reported a patient with metastatic leiomyosarcoma who received only palliative and conservative therapy and survived for 3 months after diagnosis. Radiotherapy has also been used as an adjuvant treatment along with chemotherapy [22, 23]. Finally, transarterial chemoembolization and transarterial infusion of epirubicin and carboplatin have also been reported for treatment [7, 13, 21].

PHL has aggressive metastatic potential and is usually diagnosed in situations of locally advanced or metastatic disease [5]. Surgical resection is recommended for curative treatment, while diagnosis is challenging and often delayed until reaching a large size, resulting in extremely poor prognosis [7]. Chi et al. [6] reported a median overall survival of 19 months with 1-, 2-, and 5-year survival rates of 61.2%, 41.1%, and 14.5%, respectively. They concluded that complete surgical resection with clear margin was necessary to improve survival of patients. In this case, hepatic resection was performed to confirm diagnosis and prevent tumor rupture, although multiple lung nodules, suggesting lung metastases, were found preoperatively.

## Conclusions

In conclusion, PHL is a rare malignant disease with relatively poor prognosis. To confirm PHL diagnosis, immunohistochemical analysis as well as histopathological findings is important. The preferred treatment type is surgical resection, sometimes in combination with adjuvant chemotherapy and/or radiotherapy. Further studies are required to elucidate and better understand this uncommon clinical entity and establish treatment strategies.

## Data Availability

All datasets supporting the conclusions of this article are included in this published article.

## Abbreviations

PHL: Primary hepatic leiomyosarcoma
CT: Computed tomography
MRI: Magnetic resonance imaging
PET: Positron emission tomography
αSMA: α-Smooth muscle actin
